# Population genetic analysis reveals cryptic sex in the phytopathogenic fungus *Alternaria alternata*

**DOI:** 10.1038/srep18250

**Published:** 2015-12-15

**Authors:** Jing-Wen Meng, Wen Zhu, Meng-Han He, E-Jiao Wu, Guo-Hua Duan, Ye-Kun Xie, Yu-Jia Jin, Li-Na Yang, Li-Ping Shang, Jiasui Zhan

**Affiliations:** 1 Fujian Key Lab of Plant Virology, Institute of Plant Virology, Fujian Agriculture and Forestry University, Fuzhou, Fujian, 350002, P. R. China; 2 Key Lab for Biopesticide and Chemical Biology, Ministry of Education, Fujian Agriculture and Forestry University, Fuzhou, Fujian, 350002, P. R. China

## Abstract

Reproductive mode can impact population genetic dynamics and evolutionary landscape of plant pathogens as well as on disease epidemiology and management. In this study, we monitored the spatial dynamics and mating type idiomorphs in ~700 *Alternaria alternata* isolates sampled from the main potato production areas in China to infer the mating system of potato early blight. Consistent with the expectation of asexual species, identical genotypes were recovered from different locations separated by hundreds of kilometers of geographic distance and spanned across many years. However, high genotype diversity, equal *MAT1-1* and *MAT1-2* frequencies within and among populations, no genetic differentiation and phylogenetic association between two mating types, combined with random association amongst neutral markers in some field populations, suggested that sexual reproduction may also play an important role in the epidemics and evolution of the pathogen in at least half of the populations assayed despite the fact that no teleomorphs have been observed yet naturally or artificially. Our results indicated that *A. alternata* may adopt an epidemic mode of reproduction by combining many cycles of asexual propagation with fewer cycles of sexual reproduction, facilitating its adaptation to changing environments and making the disease management on potato fields even more difficult.

Reproductive mode can have profound effects on epidemiological landscapes, population genetic dynamics, evolutionary trajectory and management of pathogens^1^,^2^ through its impacts, directly or indirectly, on the survival of spores in hazardous environments^3^, dispersal ability of the species^4^,^5^, generation, maintenance and distribution of genetic variation^6^ and efficiency of selection against unnecessary genes^7^,^8^. Pathogenic fungi have developed a wide array of reproductive strategies including asexual reproduction, sexual reproduction, and parasexual reproduction to transmit their genetic materials in natural populations^4^. Though theory assumes “two-fold” evolutionary cost^9^, sexual reproduction may have evolutionary advantages over its asexual counterpart as more genetic variation can be generated through intra-gene recombination^10^ or the rearrangement of existing genes^11^,^12^. Sexual reproduction can also increase the efficiency of natural selection against undesired alleles such as unnecessary virulence factors which may be locked in the genome of asexual species by hitchhiking^13^. Furthermore, sexual fruiting bodies of many pathogens are stress tolerant and can survive for a long time under severe conditions such as climate extremities or the absence of hosts. If the sexual progenies are wind dispersed such as sexual spores generated by many ascomycetes, long-distance gene flow is likely to occur. Increases in genetic variation, gene flow potential, and efficiency of selection against undesired alleles of sexual pathogens enhance their ability to evolve rapidly in response to the change of management strategies such as the deployment of resistant cultivars and the application of fungicides.

Sexual reproduction in fungi is usually triggered by environmental stresses or nutrition deficiency^14^ and often occurs off-season when primary hosts are not available^3^. Therefore, direct observation of the sexual stage in their life cycle is difficult for many facultative parasitic fungi, particularly for those that cannot be crossed in laboratory conditions. In such cases, population genetic analysis of spatial dynamics in selective neutral markers and mating type genes can be a powerful approach to uncover cryptic sex in plant pathogenic fungal populations^15^,^16^,^17^,^18^,^19^,^20^. Fungal populations exhibiting little or no sexual reproduction are expected to exhibit low genetic variation and a significant degree of non-random association amongst unlinked alleles. In contrast, high genetic variation and random associations amongst neutral loci indicate that the pathogen population may undergo regular sexual recombination^21^,^22^,^23^. For many heterothallic fungi, sexual reproduction is controlled by a single regulatory locus containing two alternate idiomorphs (*MAT1-1* and *MAT1-2*)^17^,^18^,^24^ and requires the interaction between individuals from distinct mating types. Heterothallic pathogens with regular cycles of sexual reproduction are expected to have the two mating types present in equal frequencies in a population as a result of frequency dependency^22^,^25^, and low genetic differentiation and no phylogenetic associations between isolates in the two mating type groups attributed to the frequent exchange of genetic material. In this study, we used population genetic approach to investigate the occurrence of cryptic sexual reproduction in the plant pathogenic fungus *Alternaria alternata*, which is associated with the early blight disease on potatoes.

Potato early blight, characterized by the formation of dark-colored spots that are necrotic in the centre with a pattern of concentric rings on leaves, is among the most destructive emerging pathogen worldwide^26^. It is a polycyclic disease and its epidemic can be rapidly built up when environments are conducive^27^. Considerable economic losses caused by potato early blight have been reported in many countries^28^. In the recent years, the frequency and scale of early blight occurrences have increased, possibly attributed to reduced nitrogen supplies, increased air temperature, withdrawal of some effective fungicides and the changes of farming systems such as the adoption of irrigation and minimum tillage for resource and environment conservations^29^. In China, early blight has been observed in all potato production areas, suggesting environmental conditions in the country are favorable for its epidemic, and the disease can occur in any stages of potato development.

Both *Alternaria alternata* and *A. solani* can cause early blight on potatoes in the world^26^,^30^,^31^,^32^,^33^. *A. alternata* produces densely turfy colonies in dark grey to black brown color on PDA plates. Its conidium is usually pale to light brown containing ~15 septa in length with numerous secondary and occasionally tertiary chains branching from apical and median cells^31^. On the other hand, *A. solani* produces densely dark grey to black colonies with sparse aerial mycelium. Its conidia usually has 9–11 transverse septa and 1–2 longitudinal septa with one long to ovoid beak^26^,^31^. In China and Europe, the majority of previous studies indicate that *A. alternata* out-competed *A. solani* though in importance the relative proportions of these two species may vary in populations^31^. Our survey from China is consistent with this result (unpublished data).

*A. alternata* is an opportunistic haploid pathogen affecting various important crops^34^. Though carrying a functional *MAT1* gene^18^, it is considered to be an asexual fungus (*Ascomycota*) because teleomorph (the sexual form) has not been observed yet in nature. Conflicting with theoretical expectation for an asexual pathogen, previous population genetic surveys revealed high genetic variation in *A. alternata* populations^26^,^35^,^36^. However, the majority of these surveys focused the descriptions of within-population variation using virulence, vegetative compatibility or genetic markers with less reliability and resolution such as RAPD. Population genetic analysis on the spatial dynamics of genetic variation in *A. alternata* using the combination of highly polymorphic neutral markers such as SSR and functional genes such as the mating type gene is scarce, particularly for *A. alternata* from potato. Knowledge from this type of analysis is important for understanding the reproductive mode and adaptive potential of the pathogen, therefore, necessary in designing an effective and sustainable disease management program for controlling this emerging disease worldwide. It is reported that *A. infectoria,* a species which occupies a basal position in the phylogeny of *Alternaria* genus, connects to a *Lewia* teleomorph^37^,^38^, suggesting that, in fact, most of the *Alternaria* species may have a sexual ancestor^39^. Furthermore, some evidence of sexual recombination was reported in *A. alternate* from citrus by sequence approach^40^. Hence, the objectives of this study were: 1) to screen specific primers for the determination of mating types in *A. alternata*; 2) to determine spatial distribution of the two mating types in *A. alternata* populations; and 3) to infer the occurrence of sexual reproduction in the field populations of *A. alternata*.

## Results

### Spatial distribution of mating type frequency in *A. alternate*

**Figure 1:**
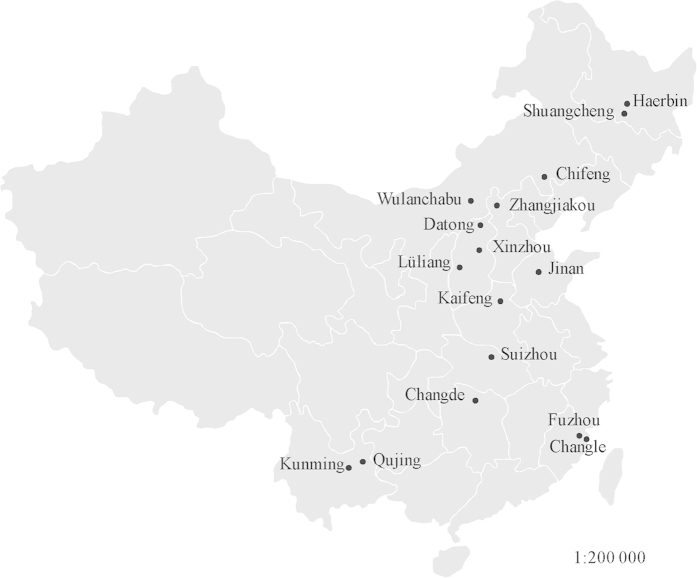
Map showing the geographic locations of the 17 *A. alternata* populations sampled from China. Adobe Illustrator Artwork 17.0 software was used to create the map.

**Figure 2:**
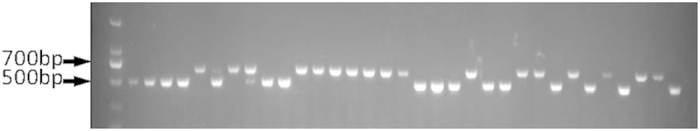
Polymerase chain reaction amplification of 33 *A. alternata isolates* from Datong with the two mating type-specific primers. The primers amplify a ~661 bp unique fragment from isolates carrying *MAT1-1* idiomorph and a 494 bp unique fragment from isolates carrying *MAT1-2* idiomorph. The first panel is a 100-bp size ladder.

A total of 725 *Alternaria alternata* isolates from different parts of China (Fig. 1) were assayed for mating type frequencies with the two pairs of primers (Table 1). All produced a unique amplification of either 661 or 494 bp, corresponding to *MAT1-1* or *MAT1-2,* respectively (Fig. 2). After clonal correction, 496 isolates were included in the analysis of mating type frequency (Table 2). Both mating types were detected in all 17 field populations sampled across China (Table 2). *MAT1-1* frequency in the field populations ranged from 0.30 in Suizhou to 0.63 in Changle with an average of 0.45 when data from the study were combined whilst *MAT1-2* frequency ranged from 0.37 in Changle to 0.70 in Suizhou with an average of 0.55 for combined data (Table 2). The hypothesis of 1:1 ratio between *MAT1-1* and *MAT1-2* frequencies was not rejected within all but one field population. Except for one case (Suizhou population), no significant differences in mating type frequency were detected amongst field populations (Table 2) and regional populations (p = 0.154).

### Genetic diversity in *A. alternata* populations

**Figure 3:**
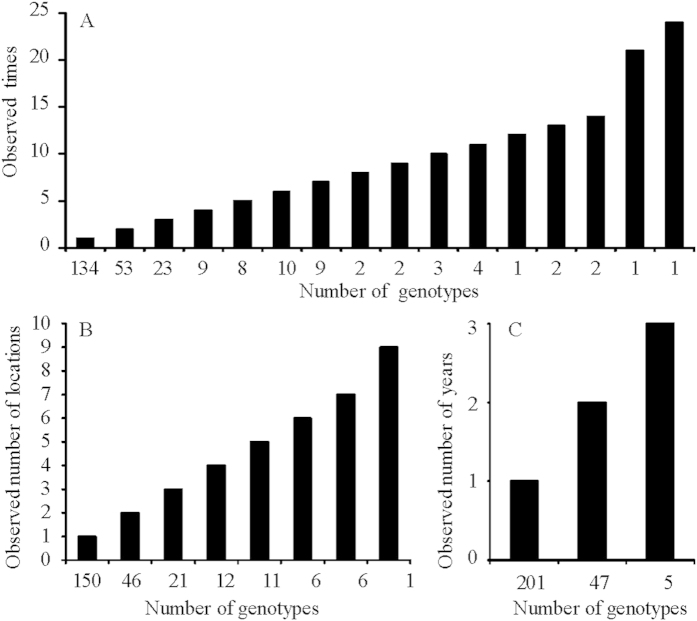
Spatial distribution of 253 multilocus genotypes in the 688 *A. alternata* isolates collected from 17 geographic locations of China between 2011 and 2013.

The isolates were assigned to 253 distinct genotypes. Among these genotypes, 134 (52.96%) were detected only once (Fig. 3A). The most common genotype was detected 24 times and the most widespread genotype was detected in 9 out of the 17 field populations (Fig. 3B). Looking at the temporal scale, 203 (80.2%), 45 (17.8%) and 5 (2.0%) out of the 253 genotypes were detected within a sampling year, in two and three sampling years (Fig. 3C), respectively. Therefore, the frequency of shared genotypes decreased from the first to third sampling year. Clonal fraction in the field populations ranged from 0.00 to 0.37 with a total clonal fraction of 0.27 in the pooled population. Correspondingly, standardized Shannon index in the field populations ranged from 0.84 to 1.00 with a grand Shannon index of 0.78 when isolates from different fields were pooled together (Table 3). Average Nei’s gene diversity in the 17 field populations ranged from 0.19 in *PAS6* to 0.79 in *PAS2* with an grand mean of 0.38 and average allele number in the 17 field populations ranged from 2.2 in *PD8* to 8.9 in *PAS2* with an grand mean of 3.7 (Table 4).

### Test for gametic equilibrium

In the 17 field populations, the hypothesis of random association was not rejected in 60.7–100% locus pairs with an average of 85.1%, and 80.3–96.7% allele pairs with an average of 88.6% (Table 3). When isolates from different fields were pooled together, the hypothesis of random association was retained in 78.4% locus pairs and 88.1% allele pairs. The observed variance of heterozygosity did not exceed either L_95_ or U_95_ values in five out of nine field populations with a sample size >30 and the corresponding I_A_ was not significantly different from theoretical expectation for random mating also in five of these populations (Table 3).

### Population differentiation and phylogenetic relatedness between *MAT1-1* and *MAT1-2* isolates

**Figure 4:**
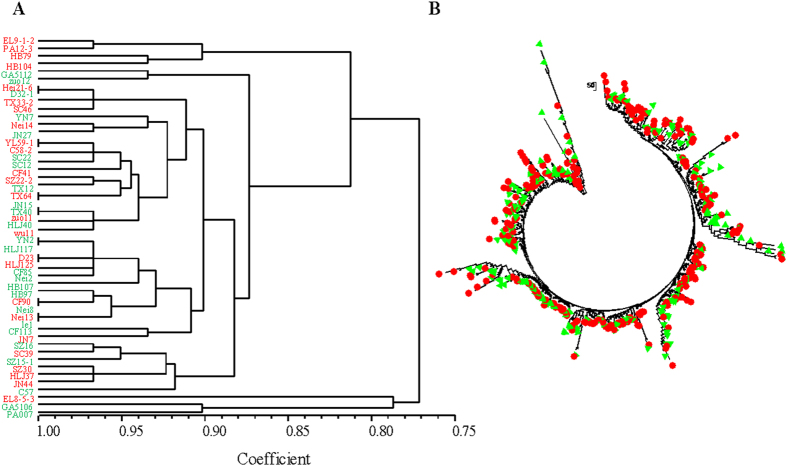
Phylogenetic relatedness of *MAT1-1* (green) and *MAT1-2* (red) isolates in the *A. alternata* population. (**A**) UPGMA tree of 25 each *MAT1-1* and *MAT1-2* isolates randomly selected from the total collection. The phylogenetic tree was reconstructed using UPGMA and displayed with NTSYS. (**B**) Neighbor joining tree of all 688 *A. alternata* isolates with definite mating type assignments. Nei’s genetic distance was calculated using GENALEX 6.5 and the phylogenetic tree was reconstructed in Mega 5.

Genetic differentiation (*G*_ST_) between the two mating type groups in the 17 field populations ranged from 0.01 to 0.31 (Table 3). Most field populations with a reasonable sample size (>16) did not have a *G*_ST_ value larger than 0.05. When all isolates from different fields were pooled together, *G*_ST_ between *MAT1-1* and *MAT1-2* isolates was 0.002. Isolates from the two mating types were randomly distributed in phylogenetic clades (Fig. 4), suggesting no genetic association between the isolates from the same mating type group.

## Discussion

Same genotypes were recovered in the *A. alternata* populations sampled from different geographic regions and years (Fig.3), consistent with the theoretical expectation for a species presumably reproducing asexually. This result suggests that infected tubers or other materials may be the main mechanisms responsible for long distance dispersal of conidia of *A. alternata* on potatoes, as observed on other crops^41^,^42^,^43^. However, clonal fraction was considerably low across populations. In addition to asexual reproduction, our results suggested that, like many presumed asexual fungi^15^, cryptic recombination might be occurring and contributing significantly to the population genetic dynamics and epidemics of this emerging potato pathogen. We have several lines of evidence that support the hypothesis of recombination in *A. alternata* populations from potatoes in China: high genotype diversity, random association among neutral markers, equal mating type frequency and no genetic differentiation between isolates of two mating type groups.

Populations without recombination will exhibit a high degree of clonality dominated by a few genotypes and low gene diversity. Though some genotypes were detected many times in our study, large genotype diversity was observed in the majority of the 17 field populations: more than 50% of genotypes were detected only once and all isolates had unique genotypes in some populations. This level of genotype diversity is comparable to some fungi with frequent sexual recombination^10^,^23^,^44^ but much higher than those reproducing primarily in clonally. For example, in an asexual potato pathogen *P. infestans*, only 49 genotypes were detected in the 531 isolates analyzed with a similar type of molecular markers^45^. High genotype diversity in *A. alternata* was also found in previous population surveys^26^,^35^,^36^.

Populations without recombination are also expected to exhibit a significant degree of non-random association among unlinked alleles. However, in the current study, all gametic equilibrium analyses indicate a low to no non-random associations amongst the SSR markers in most of the 17 field populations (Table 4). The hypothesis of random associations was retained in ~80% of allele-by-allele and ~90% of locus-by-locus comparisons. Multilocus association analysis also indicated that more than 50% (9/17) of the fungal populations displayed random association in the SSR loci. Analysis of multilocus association is strongly influenced by population sizes and result from populations with a sample size <30 is less reliable^46^. When we re-analysed the multilocus association only using the populations with ≥30 isolates, we also found the hypothesis of random mating in neutral SSR markers was retained in more than 50% of the fungal populations assayed. While many biological and evolutionary processes including genetic drift, asexual reproduction and assorting mating may lead to non-random association, the finding of random association among SSR markers strongly indicate that most of the *A. alternata* populations sampled from potatoes in China underwent regular recombination.

Genetic differentiation between isolates from *MAT1-1* and *MAT1-2* was very low when SSR variation in the two groups of the pathogen was compared, suggesting a high degree of genetic exchange between the two sub-populations of the pathogen. *G*_ST_ between two mating type groups in the majority of the 17 field populations was lower than 0.05. When the isolates from different regions were pooled to form a national population, no genetic differentiation (*G*_ST_ = 0.00) was detected between two mating type groups. In three pathogen populations (Kunming2, Changle and Qujing), moderate to high genetic differentiations were detected between the two mating type groups, but they were likely attributed to sampling error caused by small population sizes as only 5–8 genotypes were included in these populations for the analysis.

Phylogenetic analyses for the relatedness between the two mating type groups of isolates using both partial (50 isolates, Fig. 4A) and the whole set (Fig. 4B) of SSR data also indicated no genetic convergence existed, suggesting frequent exchange of genetic material through recombination homogenizing *MAT1-1* and *MAT1-2* genomes.

When combined with other measures of population genetic parameters such as genotype diversity and gametic equilibrium/disequilibrium^47^, frequency distribution of mating types in heterothallic fungi with a single *MAT* gene controlling two idiomorphs^17^ can be used to infer mating history of fungi^44^,^48^,^49^,^50^. While skewed ratio between the two mating types in a population could be caused by many biological and evolutionary events, an observed coexistence and equal frequency of both mating types would be consistent with regular occurrences of recombination. In our study, the null hypothesis of a 1:1 ratio between the two mating types was retained in all but one populations and this result remained the same regardless whether clone corrected or clone-uncorrected data were used. The observation of significant departure from a 1:1 ratio in one field population could be due to a false rejection of the null hypothesis. As the probability of the type I error for tests conducted at the P = 0.05 level is 0.05, the likely number of falsely rejected null hypotheses in our 24 tests (17 fields + 4 regions + 3 years) is 1.2.

Both meiotic recombination in sexual reproduction and mitotic recombination in parasexual reproduction could explain the observed patterns of population genetic structure in *A. alternata*. Parasexual reproduction was first described in *Aspergillus nidulans*^51^ and since then has been reported in many pathogenic fungi^40^,^52^,^53^,^54^. It first involves the fusion of hyphae (anastomosis) from different strains in the same vegetative compatibility group to form heterokaryons (heterokaryosis) and then fusion of the unlike nuclei in the cell of the heterokaryon (karyogamy) to form diploid nuclei. Homologous chromosomes in the diploid nuclei undergo synapsis and mitotic recombination (cross-over), generating recombinant chromosomes if the homologs carry different genetic information. The diploid nucleus begins to lose chromosomes gradually through cycles of mitotic divisions and return to a stable haploid state eventually. Each of these events is believed to be relatively rare^55^. For example, the frequency of chiasma formation in parasexual reproduction is much lower than that in sexual reproduction because chromosomes in the former do not pair in a regular arrangement. Furthermore, due to non-disjunctive division in diploid nuclei, parasexual reproduction is expected to form a series of aneuploid either with additional or missing copies of chromosomes, generating multiple or null amplifications by PCR amplification. In our study, we did not detect such multiple or null amplifications in any SSR amplifications of the isolates. Therefore, while we cannot exclude the possibility of parasexual reproduction, we believe the main contribution to the observed genetic variation in *A. alternata* is meiotic recombination through sexual reproduction. It is likely the sexual stage of the pathogen occurs off-season on alternate hosts, making it difficult to observe. Sexual reproduction in *A. alternata* has also been documented preciously from a small area (a single citrus grove) in North America^40^. Our results suggest sexual reproduction may commonly occur in the pathogen by using a large collection involved many population originated from wide geographical locations.

In conclusion, our results indicate that, like many eukaryotic pathogens, *A. alternata* in potatoes may have evolved to have an epidemic mode of reproduction^56^. This reproducing strategy allows the pathogen to preserve allelic combinations that are well adapted to existing hosts and environments while retain its ability to generate new allelic combinations that may offer an advantage on novel hosts and in changing environments^57^, making it more difficult to manage. In this case, using evolutionary and ecological knowledge of *A. alternata* and its hosts to design a dynamic disease management program that combine all available approaches such as host resistance, cultivar rotation, disease sanitary and fungicide is important to manage potato early blight effectively and durably^58^. The deployment of single gene resistance varieties and high risk fungicides should be done carefully because the pathogen has high evolutionary potential^59^,^60^.

## Materials and Methods

### Fungal collections

Infected potato leaves with early blight were collected at random at 1–2 meter intervals from 17 farmer fields across China (Fig. 1) between 2011 and 2013. One infected leaf was collected from each plant. To isolate fungi, infected leaves were rinsed briefly with distilled water, sterilized with 75% alcohol for 20 seconds and incubated on plates filled with 1% water-agar medium. After 24 hours, one conidium from each infected leaf was transferred to potato dextrose agar (PDA, Potato 200 g/L, Glucose 20 g/L and Agar 20 g/L) medium in a Petri dish and cultured at 25 °C for two weeks. Fungal isolates were purified by repeatedly transferring a single conidiophore to a flesh PDA plate for three times and mycelia from the third purification were then harvested for DNA extraction. Genomic DNA was extracted using plant gDNA kit (Promega Biotech. co. LTD., Beijing) according to the manufacturer’s instructions. All isolates were checked morphologically with a light microscope and molecularly by PCR amplification of ITS regions with primers 5′-TCCGTAGGTGAACCTGCGC-3′and 5′-TCCTCCGCTTATTGATATGC-3′ to confirm they were *A. alternata.*

### SSR assays of *A. alternata* populations

Genomic DNAs were amplified with the seven SSR markers previously developed for *A. solani*^61^ and one SSR marker (Ad8) previously developed for *A. dauci*^62^ in an 2720 thermal cycler (Applied Biosystems, Foster city, California) in a total reaction volume of 25 μL containing 1.0 μL of template DNA, 12.5 μL of 2X EasyTaq PCR SuperMix(-dye) (Transgen Biotech co. LTD., Beijing, China), 1.0 μL of forward primer (10.0 μM/L), 1.0 μL of reverse primer (10.0 μM/L) and 9.5 μL of sterilization water. Forward primers were synthesized by Ruiboxingke Biotech. Co. LTD. (Beijing) and labelled with different fluorescent dyes at the 5′. The program used for PCR reaction was: initially held at 95 °C for 5 min, followed by 35 cycles of 94 °C for 30 s, 57 °C (same annealing temperatures for all SSR primers) for 30 s and 72 °C for 30 s; ended with an extension step at 72 °C for 5 min. Sizes of amplification were determined by Ruiboxingke Biotechnology Co. LTD. (Beijing) using an ABI 3730XL automated DNA sequencer (Applied Biosystems, California) in which a DNA size ladder was included in each of the samples.

### Mating type determination

Mating types in *A. alternata* isolates were determined by PCR amplification of genomic DNAs with two pairs of mating type-specific primers (Table 1). The primers were designed from two complete mating type sequences of *A. alternata* (GU735423.1 and GU735413.1) downloaded from Genebank and synthesized by Ruiboxingke Biotech. Co. LTD. (Beijing). Primers A amplifies a part of the α-domain, generating a unique 661 bp fragment from the isolates carrying *MAT1-1* idiomorph and Primers B amplifies a part of the HMG-box, generating a unique 494 bp fragment from the isolates carrying *MAT1-2* idiomorph. PCR amplifications of mating types were performed using a mixture of two pairs of primers (multiplex PCR) and carried out in an 2720 thermal cycler (Applied Biosystems, Foster city, California) in 20 μL PCR reaction volume that consisted of 1 μL (10 μM) of each primer, 2 μL (2 mM) of dNTPs, 2 μL of 10 × reaction buffer, 1 unit of Taq polymerase (Transgen Biotech co. LTD., Beijing), 2 μL of genomic DNA and 9 μL of sterilized distilled water. The program used for mating type amplification was: initially held at 95 °C for 5 min, followed by 33 cycles of 94 °C for 60 s, 53 °C for 30 s and 72 °C for 30 s; ended with a final extension at 72 °C for 10 min. PCR products were separated by electrophoresis on 1.2% agarose gel at 100 V for 60 min and visualized with a G8140 Golden View I (Solarbio Science and Technology, Beijing).

### Data analyses

Alleles were assigned based on the sizes of PCR amplifications that were generated by each pair of SSR primers using GeneMaker software version 1.31 with a binning procedure. PCR amplification with an identical size generated by the same pair of primers was considered as an allele. Multilocus haplotype for each isolate was formed by joining the alleles at each SSR locus in the same order. Isolates with the same multilocus haplotype and mating type were considered as an individual member of the same clone, the asexual progeny of a genotype. In the estimation of population genetic parameters except for genotype diversity, only one member of clone was included in each population. If the clone was detected in several populations, one representative of the clone was retained for each population. All isolates (clone-corrected) were used to estimate genotype diversity.

Fungal isolates were hierarchically organized into “field” and “regional” populations. Isolates sampled from the same field were considered as the member of a field population and from different fields within the same cropping region were grouped into regional populations. There are four potato cropping regions in China: Northern Single-cropping Region (NSR), Central Double-cropping Region (CDR), Southwestern Multiple-cropping Region (SMR) and Southern Winter-cropping Region (SWR). The null hypothesis of 1:1 ratio between the two mating types within a population was evaluated by a simple χ^2^ test and heterogeneity in mating type frequency among populations was evaluated by a contingency χ^2^ test^63^ Genotype diversity was measured with standardized Shannon index^64^. The clonal fraction, defined as the proportion of fungal isolates in a population originating from asexual reproduction^23^, was calculated as 1-[(number of different genotypes)/(total number of isolates)]. Gene diversity in each SSR locus estimated in Nei’s diversity^65^ and genetic differentiation between the two mating type groups in each of the 17 field populations was evaluated by *G*_ST_^65^ using Popgen 1.32. Gametic equilibrium in the field populations was evaluated by multilocus association^46^, locus-by-locus^66^ and allele-by-allele comparisons^66^. Standard deviation of I_A_ for each population was generated by 1000 resamples of original data and its difference from theoretical expectation or random mating was evaluated by a t-test. Clone-corrected SSR data were used for these analyses. A phylogenetic tree among 25 genotypes each of the two mating types randomly selected from the total collection was reconstructed using UPGMA and displayed with NTSYS (Version 2.1, 2000, Applied Biostatistics). A further neighbor joining tree was reconstructed using SSR data of all 253 genotypes. Nei’s genetic distance^67^ in the neighbor joining tree was calculated using GENALEX 6.5 and the phylogenetic tree was displayed using Mega 5.

## Additional Information

**How to cite this article**: Meng, J.-W. *et al.* Population genetic analysis reveals cryptic sex in the phytopathogenic fungus *Alternaria alternata. Sci. Rep.* **5**, 18250; doi: 10.1038/srep18250 (2015).

## Figures and Tables

**Table 1:** Sequence, annealing temperature (T) and fragment size of PCR primers designed to amplify mating type idiomorphs of the fungal pathogen *A. alternata*.

Name	Amplification target	Sequence (from 5′to 3′)	T (°C)	Exp. size
A	*MAT1-1*	F: GAAGATTTCGTTTCAAGGCTCT	53	661
		R: TATCCCATTGACTGGACATAG		
B	*MAT1-2*	F: CATGGTCATACTTCCTGATAAC	53	494
		R: CTTCTTTCGCCGACTGTGCA		

**Table 2:** The clone corrected mating type frequencies and their homogeneity tests in 17 the field populations of *A. alternata* sampled from China between 2011 and 2013.

Population	Year	Cropping region	Sample size	Frequencies		χ^2^ test (*p-*value)	
				*MAT1-1*	*MAT1-2*	Within fields	Among fields
Changde	2012	CDR	23	0.39	0.61	0.40	
Jinan	2012	CDR	50	0.42	0.58	0.32	
Kaifeng	2012	CDR	50	0.40	0.60	0.20	
Suizhou	2012	CDR	37	0.30	0.70	0.02^*^	
Chifeng	2012	NSR	52	0.50	0.50	0.89	
Datong	2013	NSR	17	0.35	0.65	0.33	
Harbin	2012	NSR	48	0.46	0.54	0.67	
Lü´liang	2013	NSR	7	0.57	0.43	1.00	
Shuangcheng	2012	NSR	35	0.40	0.60	0.31	
Wulanchabu	2012	NSR	16	0.37	0.63	0.45	
Xinzhou	2013	NSR	10	0.50	0.50	0.75	
Zhangjiakou	2012	NSR	47	0.49	0.51	1.00	
Kunming1	2011	SMR	51	0.55	0.45	0.58	
Kunming2	2012	SMR	5	0.60	0.40	1.00	
Qujing	2011	SMR	8	0.50	0.50	0.72	
Changle	2011	SWR	8	0.63	0.37	0.72	
Fuzhou	2011	SWR	32	0.56	0.44	0.60	0.72

The hypothesis of 1:1 ratio between *MAT1-1* and *MAT1-2* within a field population was evaluated evaluaed by a simple χ^2^ test and heterogeneity in mating type frequencies among field populations and regional populations was evaluated by a contingency χ^2^ test.

**Table 3:** Tests for gametic equilibrium, genotype diversity, and genetic differentiation between *MAT1-1* and *MTA1-2* isolates in the 17 field populations of A. *alternata* collected from China in 2011-2013.

Population	Sample size^a^	No. Loci	Locus-by -locus	Allele-by -allele	Multilocus association				Clonal fraction	Shannon index	*G*_ST_ between Mating types
					S_k_^b^	L_95_^c^	U_95_^d^	*I*_*A*_(p-value)			
Changle	13	8	82.1%	90.4%	5.4287*	0.1012	2.8539	**0.21 (0.001)**	0.27	0.84	0.21
Changde	24	8	89.3%	88.1%	3.9093*	0.7771	2.7499	**0.18 (0.001)**	0.00	1.00	0.04
Chifeng	80	8	85.7%	91.4%	1.6045	0.6952	1.7239	0.06 (0.103)	0.32	0.87	0.01
Datong	26	8	89.3%	86.8%	2.6213	0.7591	2.8721	**0.14 (0.017)**	0.00	1.00	0.08
Fuzhou	48	8	60.7%	89.0%	4.2562*	0.8558	2.6766	**0.21 (0.001)**	0.16	0.9	0.05
Harbin	76	8	96.4%	94.6%	1.4569	0.6757	1.5733	0.04 (0.162)	0.35	0.85	0.02
Jinan	70	8	78.6%	90.0%	2.0734	0.7924	2.2879	0.11 (0.217)	0.29	0.89	0.01
Kaifeng	80	8	85.7%	87.4%	1.8252	0.8681	1.9766	0.10 (0.059)	0.31	0.87	0.02
Kunming1	86	8	82.1%	90.5%	1.4661	0.7102	1.7000	0.04 (0.083)	0.36	0.87	0.01
Kunming2	7	8	96.4%	81.3%	5.0525*	0.1246	3.8345	**0.17 (0.031)**	0.17	0.87	0.31
Lü´liang	9	8	89.3%	80.3%	5.3282*	0.2381	3.4558	**0.18 (0.028)**	0.22	0.86	0.08
Qujing	8	8	89.3%	90.6%	2.9877	0.2081	3.2103	0.06 (0.192)	0.00	1.00	0.14
Shuangcheng	42	8	64.3%	84.5%	2.5382*	0.6544	1.9400	**0.17 (0.007)**	0.17	0.93	0.03
Suizhou	52	8	92.9%	84.5%	3.0680*	0.7718	1.9806	**0.22 (0.001)**	0.23	0.91	0.05
Wulanchabu	23	8	100.0%	96.0%	0.9908	0.2465	1.4444	−0.02 (0.473)	0.27	0.85	0.03
Xinzhou	11	8	100.0%	96.7%	1.2500	0.2835	1.5914	−0.01 (0.397)	0.23	0.86	0.08
Zhangjiakou	70	8	64.3%	86.8%	2.8831*	0.8215	2.0667	**0.19 (0.001)**	0.32	0.87	0.08
Total	725	—	78.4%	88.1%	3.1847*	1.3344	2.6984	**0.17 (0.001)**	0.27	0.78	0.002

^a^Clone-corrected sample size was presented in Table 2

^b^Observed variance of the number of heterozygosity.

^c^Lower 95% confidence limit for the expected variance of the number of heterozygosity under null hypothesis.

^d^Upper 95% confidence limit for the expected variance of the number of heterozygosity under null hypothesis.

^*^Indicates that the hypothesis of random association among alleles were rejected at *p* = 0.05 level.

**Table 4:** Nei’s gene diversity and allele number (in parenthesis) of SSR marker loci in the 17 field populations of *Alternata alternaria*.

SSR marker locus								
Population	PAS1	PAS2	PAS3	PAS4	PAS5	PAS6	PAS7	AD8
Changde	0.61 (3)	0.77 (6)	0.45 (3)	0.45 (3)	0.64 (5)	0.15 (2)	0.45 (3)	0.47 (2)
Jinan	0.54 (4)	0.84 (9)	0.11 (3)	0.11 (3)	0.70 (6)	0.08 (3)	0.08 (2)	0.37 (2)
Kaifeng	0.53 (3)	0.83 (10)	0.08 (3)	0.08 (3)	0.63 (4)	0.13 (3)	0.08 (3)	0.44 (2)
Suizhou	0.56 (4)	0.84 (10)	0.18 (3)	0.18 (3)	0.66 (4)	0.11 (3)	0.18 (3)	0.43 (2)
Chifeng	0.45 (3)	0.86 (14)	0.14 (3)	0.14 (3)	0.68 (5)	0.10 (3)	0.14 (3)	0.14 (2)
Datong	0.60 (3)	0.86 (8)	0.29 (2)	0.29 (2)	0.56 (4)	0.21 (3)	0.29 (2)	0.48 (2)
Harbin	0.55 (4)	0.85 (12)	0.05 (2)	0.05 (2)	0.64 (4)	0.08 (3)	0.05 (2)	0.23 (2)
Lü´liang	0.20 (2)	0.69 (4)	0.20 (2)	0.20 (2)	0.49 (3)	0.37 (3)	0.20 (2)	0.20 (2)
Shuangcheng	0.54 (3)	0.86 (9)	0.14 (4)	0.14 (4)	0.74 (5)	0.17 (2)	0.14 (4)	0.13 (2)
Wulanchabu	0.45 (3)	0.79 (8)	0.00 (1)	0.00 (1)	0.57 (3)	0.08 (2)	0.00 (1)	0.08 (2)
Xinzhou	0.47 (2)	0.70 (5)	0.00 (1)	0.00 (1)	0.46 (3)	0.14 (2)	0.00 (1)	0.26 (2)
Zhangjiakou	0.34 (3)	0.83 (13)	0.11 (3)	0.11 (3)	0.60 (5)	0.32 (5)	0.11 (3)	0.13 (2)
Kunming1	0.56 (5)	0.86 (13)	0.07 (3)	0.19 (5)	0.68 (5)	0.12 (3)	0.05 (2)	0.33 (5)
Kunming2	0.00 (1)	0.72 (4)	0.28 (2)	0.28 (2)	0.61 (3)	0.00 (1)	0.28 (2)	0.44 (2)
Qujing	0.00 (1)	0.75 (5)	0.41 (3)	0.22 (2)	0.41 (3)	0.22 (2)	0.22 (2)	0.47 (2)
Changle	0.73 (4)	0.64 (4)	0.74 (5)	0.74 (5)	0.00 (1)	0.46 (2)	0.74 (5)	0.40 (2)
Fuzhou	0.73 (6)	0.80 (8)	0.69 (5)	0.68 (5)	0.44 (5)	0.54 (3)	0.69 (5)	0.52 (3)
Average	190–208	235–295	189–201	197–218	238–256	216–232	156–168	123–141
Range*	190–208	235–295	189–201	197–218	238–256	216–232	156–168	123–141

^*^Range of fragment size.
